# Fahr Syndrome: When Dysarthria Doesn’t Mean a Stroke

**DOI:** 10.7759/cureus.50616

**Published:** 2023-12-16

**Authors:** Daniela Casanova, Ana L Ferreira, Ana Sá, Isabel Trindade, Jorge Cotter

**Affiliations:** 1 Internal Medicine, Hospital da Senhora da Oliveira - Guimarães, Guimarães, PRT; 2 Internal Medicine, Hospital da Senhora da Oliveira, Guimarães, PRT; 3 Internal Medicine, Hospital Senhora da Oliveira - Guimarães, Guimarães, PRT

**Keywords:** idiopathic basal ganglia calcification, phosphocalcium metabolism, fahr syndrome, stroke, dysarthria

## Abstract

Fahr syndrome is a rare neurodegenerative disorder, characterized by calcium deposition in the brain. It is usually associated with phosphocalcium metabolism disorders, like hypoparathyroidism, or with genetical predisposition, as seen in Fahr disease. Given the wide array of differential diagnoses medical awareness should be emphasized to prompt diagnosis and management.

In this case, we depict a classical presentation of Fahr syndrome, highlighting the differential diagnosis with stroke given the similar clinical signs and symptoms, although pointing out the distinct radiological presentation that raises clinical suspicion for this entity.

## Introduction

Fahr syndrome is a rare neurodegenerative disorder (prevalence of < 1/1.000.000), characterized by calcium deposition on basal ganglia and other regions of the brain, with cellular death associated [1-2]. Its etiology may derive either from phosphocalcium metabolism disorders, with hypoparathyroidism at the top of the differential diagnosis list, as well as from genetic abnormalities, thus designated as Fahr disease [3-4]. The treatment of this nosologic entity is mostly symptomatic but the absence of a rapid diagnosis, given its progressive installment, leads to life quality degradation [5].

## Case presentation

A 53-year-old man presented to the emergency department (ED) with worsening dysarthria and an acute onset of diminished strength in his right upper limb. The patient reported a five-year history of chronic speech disturbances with a gradual onset, particularly affecting the dictation of certain words and sentences. The dysarthria had worsened on the day of the presentation, rendering him unable to speak properly and prompting him to seek care in the ED. Additionally, the patient mentioned reduced strength in the right upper limb, mainly in the palmar region, preventing him from holding small objects. This decrease in strength had an acute onset on the same day. 

**Figure 1:**
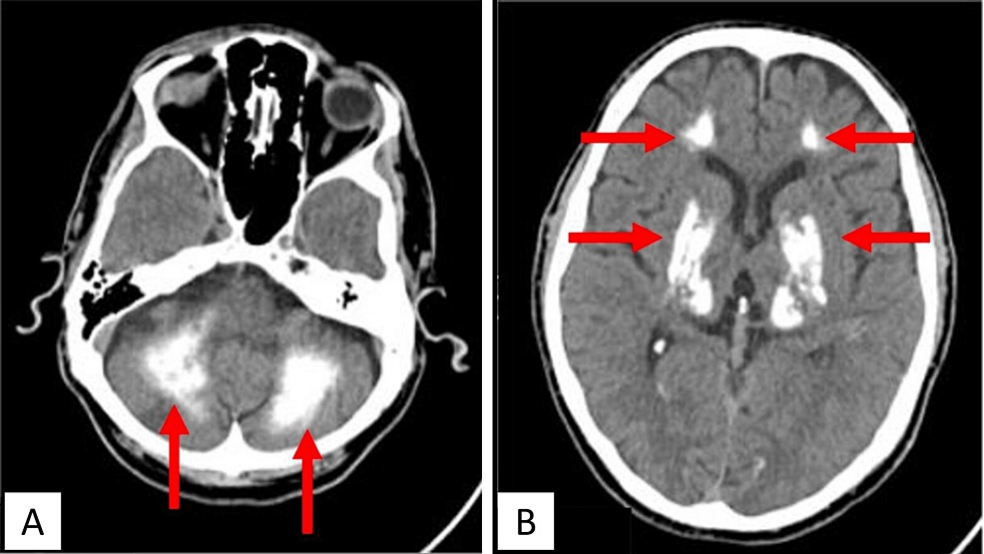
Cranial computed tomography Red arrows show multiple calcifications

On neurologic examination, moderate dysarthria was noted, with no discernible difference in limb strength, and a rating of five out of five on the scale. A cranioencephalic computed tomography was performed and showed multiple calcifications in semi-oval centers, radiated coronas, basal ganglia, thalamus, brainstem, and cerebellar hemispheres (Figures 1a, 1b). 

The patient was hospitalized for etiological clarification, MRI excluded ischemic stroke, and no other risk factors were found besides dyslipidemia. The analytic study had no alterations regarding phosphocalcium metabolism (Table 1). 

**Table 1:** Blood analysis

Blood analysis	Value	Reference	
Hemoglobin	14.9g/dL	14.0-18.0	
Hematocrit	40.5%	41-53	
Mean corpuscular volume	81.7fL	83-103	
Mean corpuscular hemoglobin	30.4pg	28-34	
Mean corpuscular hemoglobin concentration	35.6g/dL	32-36	
White blood cells	6.4x10^3^/uL	4.8-10.8	
Neutrophils	4.4x10^3^/uL	1.8-7.7	
Lymphocytes	2.0x10^3^/uL	1.0-4.8	
Platelets	280x10^3^/uL	150-350	
Creatinin	0.72mg/dL		0.70-1.30
Sodium	143mEq/L		135-146
Potassium	4.1mEq/L		3.5-5.1
Calcium	8.5mg/dL		8.3-10.6
Phosphorus	4.1mg/dL		2.5-4.9
Magnesium	2.3mg/dL		1.6-2.6
Vitamin D	76ng/mL		30-100
Parathyroid hormone (PTH)	57pg/mL		18.5-88.0
Albumin	3.8g/dL		3.4-5.0

The patient started language therapy during hospitalization and was referred to consult internal medicine and neurology to continue surveillance every six months.

## Discussion

This clinical case presents the typical clinical and radiological features of Fahr disease, a rare disorder characterized by calcium deposition on the brain without disorders in phosphocalcium metabolism [1-2]. It typically presents classically around the age of 40-50 [1-2], like in our case.

The most common differential diagnoses are iatrogenic parathyroidectomy, radiotherapy, local infiltration, vascular events, and metastasis. Stroke may arise as one possible hypothesis given the similar clinical signs and symptoms; however, some chronicity is usually present from the onset, allowing for differentiation, as demonstrated in our case [3-4]. 

As a rare neurodegenerative disorder, little is written about it, and treatment of this nosologic entity is mostly symptomatic, with a progressive evolution leading to a degradation in the quality of life [5]. Typically, at this stage of onset, the syndrome is characterized by seizures, and psychiatric manifestations are disturbing, while dementia and movement disorders may take more time to develop [6], which differs somewhat from what was observed in our case.

Although it is an incurable disease, new and improved strategies focused on symptomatic relief and prevention through genetic counseling are enhancing the quality of life for patients [7]. 

## Conclusions

In conclusion, this case serves as a classical presentation of Fahr's disease, with a specific emphasis on its distinctive imaging features. By presenting this typical scenario, we aim to familiarize healthcare professionals with the presentation of Fahr's disease, ensuring that its recognition in emergency departments becomes more commonplace.

It is crucial to underscore that this condition, as evidenced in this case, is an incurable condition that significantly impacts patients' lives, leading to disability. This highlights the imperative for further studies focused on treatment strategies and the accumulation of evidence surrounding this rare disease. Increased research efforts are essential to better understand this disorder, ultimately paving the way for improved patient outcomes and management.
